# Comprehensive Review on Application of FTIR Spectroscopy Coupled with Chemometrics for Authentication Analysis of Fats and Oils in the Food Products

**DOI:** 10.3390/molecules25225485

**Published:** 2020-11-23

**Authors:** Abdul Rohman, Mohd Al’Ikhsan B. Ghazali, Anjar Windarsih, Irnawati, Sugeng Riyanto, Farahwahida Mohd Yusof, Shuhaimi Mustafa

**Affiliations:** 1 Center for Excellence Institute for Halal Industry and Systems (IHIS), Universitas Gadjah Mada, Yogyakarta 55281, Indonesia; 2 Department of Pharmaceutical Chemistry, Faculty of Pharmacy, Universitas Gadjah Mada, Yogyakarta 55281, Indonesia; sugeng_riyanto@ugm.ac.id; 3 Islamic Civilisation Academy, Universiti Teknologi Malaysia, Kuala Lumpur 54100, Malaysia; alikhsan.kl@utm.my (M.A.B.G.); farahwamy@utm.my (F.M.Y.); 4 Research Division for Natural Product Technology (BPTBA), Indonesian Institute of Sciences (LIPI), Yogyakarta 55861, Indonesia; anjarwindarsih2@gmail.com; 5 Faculty of Pharmacy, Halu Oleo University, Kendari 93232, Indonesia; irnawati.vhina@gmail.com; 6 Halal Products Research Institute, Universiti Putra Malaysia (UPM), Selangor, Darul Ehsan 43400, Malaysia; shuhaimi@biotech.upm.edu.my

**Keywords:** vibrational spectroscopy, FTIR spectroscopy, multivariate data analysis, edible fats and oils, authentication analysis

## Abstract

Currently, the authentication analysis of edible fats and oils is an emerging issue not only by producers but also by food industries, regulators, and consumers. The adulteration of high quality and expensive edible fats and oils as well as food products containing fats and oils with lower ones are typically motivated by economic reasons. Some analytical methods have been used for authentication analysis of food products, but some of them are complex in sampling preparation and involving sophisticated instruments. Therefore, simple and reliable methods are proposed and developed for these authentication purposes. This review highlighted the comprehensive reports on the application of infrared spectroscopy combined with chemometrics for authentication of fats and oils. New findings of this review included (1) FTIR spectroscopy combined with chemometrics, which has been used to authenticate fats and oils; (2) due to as fingerprint analytical tools, FTIR spectra have emerged as the most reported analytical techniques applied for authentication analysis of fats and oils; (3) the use of chemometrics as analytical data treatment is a must to extract the information from FTIR spectra to be understandable data. Next, the combination of FTIR spectroscopy with chemometrics must be proposed, developed, and standardized for authentication and assuring the quality of fats and oils.

## 1. Introduction

Edible fats and oils are considered as important components of the food products. Besides, in the recent year, animal fats and vegetable oils are considered as economic sources to be used not only in the food but also in oleochemical and pharmaceutical industries. Nutritionists recommended that people consume vegetable oils as a source of essential fatty acids such as oleic, linoleic, and alfa-linolenic acids and fat-soluble vitamins A, D, E, and K needed by human metabolism [1,2]. There are variety food products that are mostly composed of fats such as margarines and shortenings as well as vegetable oils such as cooking oils, salad or salad dressings, and other foods containing fats and oils. The quality of food products containing fats and oils is dependent on their qualities, including the authenticity, purity, and some intrinsic quality parameters [3]. Based on data retrieved from Scopus at www.scopus.com, using food adulteration as keywords, edible oils and fats are one of the most frequently adulterated food components. Valdes et al. reported that the adulteration of fats and oils ranked the third cases to be adulterated per product category with a percentage of 11%. The most adulterated products are meat and meat products accounting for 27%, followed by fish-based food products accounting for 13% [4].

With the advance of science and technology in food science, the practice of adulteration in food products, including fats and oils, has been a concern by all parties including consumers, producers, and regulators since the beginning of civilization. The adulteration practice decreases the quality of food products and affects adverse effects in human health [5]. The health-related problems may not be big issues in the adulteration practice of fats and oils, because the oil adulterants are typically edible [6]; however, in certain communities with allergenic reactions, the adulteration of vegetable oils has caused serious health problems such as Spanish olive oil syndrome [7]. Fats and oils could be adulterated in several ways: (1) the substitution of high-quality fats and oils with lower ones such as the substitution of the highest grade olive oil, namely extra virgin olive oil (EVOO), with lower grade of olive oil or pomace olive oil; (2) the dilution of expensive oils with cheap oils such as dilution of EVOO with palm oil and corn oil; (3) the mislabeling of high-priced oils such as palm oil labelled with EVOO; (4) geographical origin. Therefore, the authentication analysis of fats and oils is very important to assure that the fats and oils are authentic and free from adulteration practice [8]. The authentication analysis of fats and oils is the analytical procedure verifying that the studied fats and oils were compliant with its label description. During authentication analysis, analysts can employ traditional analysis such as determination of constant parameters including saponification and iodine numbers, organoleptic analysis, or modern methods using sophisticated instruments such as liquid chromatography equipped with mass spectrometer detectors [9].

Numerous analytical methods mainly based on spectroscopic, chromatographic, and molecular techniques have been applied and reviewed for authentication analysis of high quality fats and oils as well as food products with fats and oils as main components [9], including chromatography combined with chemometrics and metabolomic studies [10], near infrared spectroscopy combined with chemometrics [11], Fourier transform mid infrared (FT-MIR) spectroscopy, DNA-based methods such as polymerase chain reaction (PCR) [12], liquid chromatography through triacylglycerol compositional data in combination with chemometrics [13,14], electronic nose and electronic tongue [15], and differential scanning calorimetry [16]. Among these methods, FT-MIR, typically simplified to FTIR in combination with chemometrics, was the most reported method for the authentication of fats and oils due to the nature of FTIR spectra as fingerprint analytical tools. FTIR spectra are complex in nature and not easy to interpret. However, using multivariate data analysis or chemometrics, FTIR spectra could be transformed and extracted into chemical information according to the analytical purposes including classification and quantification. The chemometrics classification offered the ease clustering between the authentic edible fats and oils and adulterated ones, while chemometric quantification such as multivariate calibration provides the predictive ability of adulteration levels [17,18,19].

Some reviews on authentication of fats and oils published in the scientific literature exists. However, the previously published review articles reported all analytical methods or only reported the specific edible fats and oils such as reviews on the authentication analysis of Camellia oil [20]. Esteki et al. have reviewed on application of chromatography in combination with multivariate data analysis for authentication of food products including fats and oils [10]. Bosque-Sendra et al. [14] review the authentication of fats and oils using chromatography and chemometrics from triacylglycerol compositional data. Nunes [3] used vibrational spectroscopy and chemometrics for authentication and evaluation of intrinsic quality parameters of edible oils and fats, but the oils discussed are only certain edible fats and oils. In addition, the review on the combination of FTIR spectroscopy and chemometrics for authentication of other edible fats and oils have been also reported such as FTIR spectroscopy for authentication of fruits and vegetables [21], infrared spectroscopy for authentication of dairy products [22], species authentication and geographical origin discrimination of herbal medicines [23], and the review of vibrational spectroscopy including IR spectroscopy for the authentication of animal and vegetable food products with high fat content [4].

Our group have reviewed the authentication of edible fats and oils including authentication of olive and virgin coconut oils [24,25] and the authentication of food products from non-halal fats (lard) using different methods [26,27]. However, the methods used in these reviews are not only specific using FTIR spectroscopy but also other chemical and biological methods. Besides, the object to be reviewed is specific for certain fats and oils. For example, Rohman and Che Man [26] have used vibrational spectroscopy for the authentication of functional oils, namely olive oil and coconut oils, and did not cover all edible oils or all functional oils; therefore, in this review, the comprehensive reports on the application of infrared spectroscopy combined with chemometrics for the authentication analysis of fats and oils are presented. Specifically, the objectives of this review were (1) to update the use of vibrational spectroscopy with emphasis on FTIR spectroscopy for the authentication analysis of fats and oils in a simple mixture from previously published articles and (2) to highlight the use of FTIR spectroscopy and chemometrics for the authentication of fats and oils extracted from food products.

## 2. Methods

While preparing this review, some guidelines from scientific literature were followed [28,29,30,31,32]. The study began by collecting information from original articles, review articles, or reports, which appear in several databases including Scopus, PubMed, Web of Science, and Google Scholar. The keywords used during literature searching and literature selection either separately or combined were “FTIR” or “infrared spectroscopy”, “vibrational spectroscopy”, “adulteration analysis”, “authentication analysis”, “chemometrics” or “multivariate data analysis”, “principal component analysis”, “discriminant analysis”, “partial least square”, “partial least square-discriminant analysis”, “principal component regression”, “fats”, and “oils”. The abstracts of the papers were reviewed to select the suitable papers for this review. The inclusion criteria of selected papers were (1) studies regarding authentication analysis of edible and functional oils for quality assessment and adulterant detection using FTIR and NIR spectroscopy between 2000 and 2020; (2) studies on analysis of fats in fats-based products and food products for authentication purposes using FTIR spectroscopy between 2000 and 2020; (3) studies employing chemometrics of multivariate analysis for authentication of oils and fats; (4) all papers written in English language.

## 3. FTIR Spectroscopy and Chemometrics for Authentication of Fats and Oils

Fourier-transform infrared (FTIR) spectroscopy is one of vibrational spectroscopies based on interaction between functional groups present in the analyzed samples with electromagnetic radiations resulting the vibrational energy levels. In food industry, FTIR spectroscopy, especially combined with attenuated total reflectance (ATR) and chemometric software, has emerged as rapid non-destructive and reliable techniques for authentication analysis [33]. This technique is capable of qualitatively and quantitatively discriminating the authentic and adulterated foodstuff based on FTIR spectral characteristics [34]. The infrared (IR) region of electromagnetic radiation covers wavenumbers (1/λ) ranging from 14,000–50 cm^−1^, which can be divided into three regions, namely near IR corresponding to region of 1/λ 14,000–4000 cm^−1^, mid IR region covering 1/λ 4000–400 cm^−1^, and far IR region at 1/λ of 400–50 cm^−1^. Among these three regions, MIR is the most widely applied for authentication analysis of fats and oils; therefore, in this review, we focused only on the MIR region. Recently, some reviews on the application of FTIR spectroscopy combined with chemometrics existed in different fields including biopharmaceuticals [35], halal food authentication analysis [36], authentication of meat and meat-based food [37], discrimination and authentication of herbal products [38], and authentication analysis of biomolecules in biomedical fluids [39]. FTIR spectra obtained during authentication analysis are complex and difficult to interpret; fortunately, with the development of chemometrics software and computer technology, the problems can be solved.

Chemometrics is the application of statistics and mathematics in chemical data, which can be FTIR spectra, chromatograms, and others. The International Chemometrics Society has defined chemometrics as the science of relating chemical measurements made on a chemical system to the property of interest (such as concentration) through the application of mathematical or statistical methods [40]. The successful application of FTIR spectroscopy for authentication of edible fats and oils was supported by development of chemometric software. Some user-friendly software was commercially provided by some manufacturers such as Minitab^®^ (State College, Pennsylvania, USA), Unscrambler^®^ (Camo Analytics, Molndal, Sweden), SIMCA^®^ (Sartorius, Gottingen, Germany), and MATLAB^®^PLS_Toolbox (Mathworks, MA, USA). The purpose and characteristics of each software have been reviewed by Brereton et al. [41]. Besides, some open access chemometrics software were also available such as the software packages of R factoextra and FactoMineR, which are successfully used for chemometrics application in fats and oils authentication [42].

Chemometric methods in data analysis are pervasive and important toward the decision-making and problem-solving processes. Chemical analysis deals with complex mixtures, compounds, and their properties, which are often very complicated to be analyzed. Development of computerized laboratory automation has led to the advancement of chemical data analysis with the aid of chemometric methods as tools for analyzing and structuring the data. Chemometric methods are capable of solving problems involving classifications of different samples and determining the properties of a chemical compound [43,44].

The variables used for creating multivariate models during the authentication analysis of fats and oils were absorbance values in the selected region of FTIR spectra. FTIR measured fats and oils in a whole pattern unlike the HPLC and GC methods, which analyze part of compounds such as triacylglycerol and fatty acid, respectively. The FTIR region represents the vibration of functional groups present in studied oils and fats. Certain regions correspond to vibrations of particular functional groups in a compound, which can be stretching or bending vibrations. Adulteration of oils and fats is difficult to recognize visually, because the spectra of adulterated oils and fats remain the same as the authentic one. However, there must be changes in absorbance values in certain regions because of the different compositions in adulterated samples. Because of the addition with other oils and fats, the compositions of compounds in adulterated samples will obviously change. Some compounds could be in higher concentration, while others are in a lower concentration. Therefore, selected FTIR fingerprints used in multivariate models correlated with the changes in certain compounds in samples. As a consequence, there will be differences in absorbance values or wavenumber shifting of vibration [27,33].

In the authentication of fats and oils, various chemometric techniques are commonly used to analyze the complex chemical data, namely (1) pre-processing spectra, (2) chemometric classification analysis, and (3) chemometrics for quantitative analysis facilitated with multivariate calibration. Some data pre-processing, such as mean centering, Savitzky–Golay-based derivatization, standard normal variate, baseline corrections, signal correction and compression, spectra normalizations, and multiplicative correction are used to obtain optimum result. The detailed chemometrics applied in classification and quantitative analysis were reviewed by Rohman and Windarsih [36].

There are two types of chemometrics, namely chemometrics of pattern recognition and chemometrics of multivariate calibration. Chemometrics of pattern recognition consist of exploratory analysis including principal component analysis (PCA) and cluster analysis (CA) as well as the classification analysis including discriminant analysis (DA), partial least square discriminant analysis (PLS-DA), and orthogonal projection to latent structures discriminant analysis (OPLS-DA). Chemometrics of exploratory analysis is aimed for sample differentiation, whereas chemometrics of classification are aimed at sample classification. PCA and CA are categorized as unsupervised pattern recognition. In PCA, original variables used for creating models are reduced to just several variables called principal components (PC), having large variations representing the original variables. The PCs are responsible for samples grouping by searching the differences between variables. For cluster analysis, the grouping is performed based on similarities of variables. Meanwhile DA, PLS-DA, and OPLS-DA are categorized as supervised pattern recognition. DA classifies samples by minimizing class ratio between membership and maximizing class ratio within membership. PLS-DA works by finding the variables both in X and Y matrix responsible for the classification of established classes, while OPLS-DA searches for orthogonal variables capable of class differentiation [36].

On the other hand, chemometrics of multivariate calibration are used for multivariate quantitative analysis such as predicting concentration of oil adulterants. Partial least square (PLS) and principal component regression (PCR) are widely used for preparing the correlation between actual values of fats and oils with the predicted values using certain variables. PLS searches latent variables either in the actual matrix or in the predicted matrix to create multivariate regression, whereas PCR regression uses principal components to create a regression model. The regression curve is evaluated using some statistical parameters, namely R^2^ (coefficient of determination) for accuracy evaluation as well as root mean square error of calibration (RMSEC) and root mean square error of prediction) (RMSEP) for evaluation the precision of analytical methods used during authentication analysis [33,34].

## 4. Application of Vibrational Spectroscopy for Authentication of Fats and Oils

Table 1 shows the comprehensive review related to application of FTIR spectroscopy and chemometrics for authentication of higher edible fats and oils from the lower fats and oils, compiled from the year 2000 to 2020.

### 4.1. Olive Oil

FTIR spectroscopy in combination with chemometrics has been widely used for authentication of olive oils with different grades. FTIR spectroscopy in combination with cluster analysis (CA) and partial least square discriminant analysis (PLS-DA) has been applied for authentication of olive oils according to its region (Morocco). FTIR spectra were subjected to derivatization based on Savitzky–Golay algorithm for reducing the noise and extracting the largest number of analytical information from FTIR spectra. Olive oil samples from different locations in Morocco were divided into calibration and prediction sets. Dendrogram, as CA results using the variable of absorbance values of the whole IR region (4000–650 cm^−1^), could make groupings among samples according to their location. PLS-DA using the same variables was capable of classifying olive oil samples from the same regions, picked in different times, and unknown samples according to their classes [45].

Vibrational spectroscopies (FT-MIR and Raman) were applied for the classification of olive oil (OO) and OO added with vegetable oils for authentication studies. Samples (OO) with different qualities (EVOO, VOO, blend of virgin, and refined OO and POO/pomace olive oil) were used. Vegetable oils used as adulterants were hazelnut, peanut, canola, safflower, sunflower, flax, corn, palm, seeds, sesame, soybean, wheat, and grapeseed oils. The chemometric techniques exploited were PCA for reducing the variables, k-nearest neighbors (kNN), PLS-DA, one-class partial least squares (OCPLS), support vector machine classification (SVM-C), and SIMCA for classification, as well as PLSR for quantification. All pure samples were subjected to transesterification and the transesterified fraction were scanned using FTIR and Raman. PCA using four PCs was used for determining wavenumbers region for chemometric analysis, and finally, FTIR spectra at the combined wavenumbers region of 3100–2700, 1800–1600, and 1205–1080 cm^−1^ and Raman spectra at wavenumbers of 950–650 cm^−1^ were preferred for analysis. PLS-DA and SVM-C techniques offered good classification with accuracy levels of 100% (olive oil samples) and 92% (other vegetable edible oils). For quantitative analysis, FTIR combined with PLSR offered better results than Raman, as indicated by high R^2^ and low values of RMSECV (root mean square error of cross validation) [46].

Rohman et al. [47] also employed FTIR spectroscopy combined with PLS calibration and DA for prediction and classification of oil adulterants (canola oil, Ca-O) in EVOO. The prediction of CaO in EVOO was done by optimizing the wavenumbers capable of providing the best of performance characteristics of the PLS model, which related to the correlation between actual values of CaO and the calculated values using FTIR spectroscopy. PLS regression using the combined wavenumbers region of 987–1200 and 2985–3028 cm^−1^ were used for the prediction of Ca-O in EVOO having coefficient of determination (R^2^) of 0.99, RMSEC of 0.108% (*v*/*v*), and RMSEP of 1.52% (*v*/*v*). DA using the same variable can also classify between pure EVOO and EVOO adulterated with Ca-O with accuracy levels of 98%.

All authors used the different FTIR spectra parameters including wavenumbers region, spectral treatments (derivative or normal spectra), and chemometrics techniques during authentication of EVOO and other edible fats and oils, as detailed in the next section. The edible fats and oils are metabolites extracted from corresponding plant and animals having different composition, and as a consequence, FTIR spectra of fats and oils are slightly different; therefore, the optimization of FTIR and chemometrics condition is a must before performing authentication analysis [3].

### 4.2. Virgin Coconut Oil

Virgin coconut oil (VCO) is an emerging functional oil in the fats and oils industry due to its capability to provide some beneficial health effects. Without any chemical refining, bleaching, and deodorizing during preparation, VCO could retain some bioactive compounds such as phenolics responsible to biological activities including antioxidant, anti-bacterial, anti-inflammatory, immunomodulatory, anti-hyperlipidemia, anticancer, and antidiabetic, as reviewed by Rohman et al. [6]. VCO has a commanded high-price value in the fats and oils industry; hence, VCO can be target of adulteration with low priced oils. Recently, simple and rapid attenuated total reflectance (ATR)-FTIR spectroscopy combined with PCA and data-driven soft independent modeling of class analogy (DD-SIMCA) were applied for checking the authenticity of VCO from other vegetable oils, namely canola (CNO), corn (CO), sunflower (SFO), and soybean (SO). During analysis, ATR-FTIR spectra were subjected to mean centering and derivation. PCA using the variable of absorbance values in the whole mid IR region (4000–650 cm^−1^) was used, and the results confirmed that both pure and adulterated VCO with CNO, CO, SFO, and SO could be classified. The PCA scores presented a clear separation between pure and adulterated coconut oil samples. In addition, the sensitivity (fraction of all the target samples that are correctly classified as target samples) and specificity of developed model (DD-SIMCA) were evaluated using formula:

(1) $$\mathrm{Sensitivity}\;=\;100\;\times \;\frac{\left(\mathrm{TP}\right)}{\left(\mathrm{TP}+\mathrm{FN}\right)}$$

(2) $$\mathrm{Specificity}\;=\;100\;\times \;\frac{\left(\mathrm{TN}\right)}{\left(\mathrm{TN}+\mathrm{FP}\right)}$$

TP is true positive (the number of target samples attributed as target samples); FN is false negative (the number of target samples attributed as non-target samples); TN is true negative (the number of non-target samples attributed as non-target samples), and FP is false positive (number of non-target samples attributed as target samples) [48].

The proposed methodology (ATR-FTIR spectroscopy combined with DD-SIMCA) was capable of confirming the authenticity of VCO and capable of detecting the adulteration practice of VCO with all tested oils in a concentration range of 10–40%. In addition, the method was suitable to identify the four adulterant oils studied with sensitivity levels of 88–100% and specificity levels of 96–100% [49].

### 4.3. Red Fruit Oil

Red fruit oil (RFO) is oil obtained from the extraction of red fruit (*Pandanus conoideus* Lam) having special shape, i.e., oval with bright maroon red color. Red fruit widely distributed at Papua Island, namely Papua (Indonesia) and Papua New Guinea, having 55–100 cm long, 10–15 cm diameter, and 2–3 kg weight [78]. RFO is red in color due to high contents of carotenoids and is reported to have several biological activities including treating several degenerative diseases such as cancer, arteriosclerosis, rheumatoid arthritis, stroke, and diabetes mellitus [79]. The price of RFO in Indonesian market is 10–15 times higher than common vegetable oils; therefore, RFO is subjected to adulteration by unethical sellers to get economical profit. FTIR spectroscopy is an effective method for authentication of RFO due to its capability to differentiate FTIR spectra of RFO with other fats and oils. PCA is used to seek vegetable oils having similar FTIR spectra profiles to RFO, and based on a score plot of PC1 and PC2, RFO was located on the negative side close to canola oil (CaO) and rice bran oil (RBO). Therefore, CaO and RBO were selected as oil adulterants to RFO. Classification and quantification of RFO adulterated with CaO and RBO were assisted with chemometrics of DA and PLSR. DA using wavenumbers of 1200–1050 cm^−1^ could discriminate pure RFO and RFO mixed with oil adulterants (CaO and RBO) with no misclassification reported or in other words with accuracy levels of 100%. Detailed investigation revealed that RFO has a closer distance to CaO than RBO. This is not surprising, because RFO was more similar to CaO than RBO in terms of fatty acid compositions and FTIR spectra. The wavenumbers of 1200–1050 cm^−1^ was also used for quantification of CaO in RFO. The PLSR model for the correlation between actual and predicted values of CaO in RFO resulted in R^2^ > 0.999, with an intercept of −0.081, RMSEC of 0.812% (*v*/*v*), RMSEP of 1.05% *(v*/*v*), and RMSECV of 2.28%. In addition, the levels of RBO in RFO were better quantified at combined wavenumbers of 1207–1078 and 1747–1600 cm^−1^, resulting in R^2^ > 0.99 in calibration and validation models with low errors. Therefore, it can be concluded that FTIR spectroscopy combined with DA and PLSR is an accurate and precise method for the authentication of RFO [80].

### 4.4. Avocado Oil

Avocado oil (AVO) is oil extracted from pulp of avocado and is considered a functional oil, having a high price in the market. AVO is reported to have health-promoting effects including reducing plasma cholesterol and cardiovascular diseases due to its high content of unsaturated fats and phytosterols [81]. The authentication of AVO with other lower priced oils, such as palm oil (PO) and canola oil (CaO), is needed to assure the quality of AVO from adulteration practice. Two multivariate calibrations of PLSR and PCR were optimized using selected wavenumbers. PLSR at the wavenumbers region of 1260–900 cm^−1^ provided the best calibration models for analysis of AVO adulterated with PO, having the highest R^2^ of 0.999, RMSEC of 0.80%, and RMSEP of 0.79%. In addition, R^2^ value, RMSEC, and RMSEP values obtained for AVO adulterated with CaO at combined wavenumbers of 3025–2850 and 1260–900 cm^−1^ were 0.9995, 0.83, and 0.64%, respectively [82].

### 4.5. Sesame Oil

Sesame oil (SEO), extracted from sesame seed (*Sesamum indicum* L.), is one of the most valuable oils due to its bioactive properties containing high levels of polyunsaturated fatty acids, mainly oleic and linoleic acids. SEO has a pleasant odor and mild taste with an excellent stability due to natural antioxidants contained such as sesamin, sesamolin, and sesamol [83]. Therefore, SEO commands a high price in the market, and as a consequence, SEO is subjected to be adulterated with vegetable oils having lower price than SEO, namely hazelnut (HZO), canola (CNO), and sunflower oils (SFO). ATR-FTIR spectroscopy combined with PCA, CA, and PLSR was used for the classification and quantification of SEO adulterants. SEO was adulterated with HZO, CNO, and SFO in the concentrations ranging from 1–50%. Dendrogram of CA using variable of absorbance values at combined wavenumbers region of 1267–1209, 1121–1045, and 896–814 cm^−1^ resulted in the clear differentiation and classification of pure SEO and SEO adulterated with vegetable oils. PCA using first and third principle components was successfully used for the classification of pure SEO, HZO, CNO, SFO, and SEO adulterated with these vegetable oils. The difference in saturated and unsaturated fatty acid composition was resulted in FTIR spectra of adulterated SEO. PLSR using absorbance values at combined wavenumbers region of 1267–1209, 1121–1045, and 896–814 cm^−1^ provided true and precise results for the simultaneous quantitative analysis of SEO adulterants, as indicated by R^2^ > 0.95 for all samples with low RMSEC and RMSEP [84].

### 4.6. Black Cumin Seed Oil

Black cumin seed oil, also known as *Nigella sativa* oil (NSO), is an oil extracted from the seed of *N. sativa* [66]. NSO is widely used as traditional medicine due to some of the bioactive compounds it contains. NSO contained essential fatty acids, tocopherols, phytosterols, polyphenols, and thymoquinone, an active compound believed to be responsible in many health beneficial properties [66]. NSO has a high price in the fats and oils industry; therefore, NSO can be adulterated with low-price oils such as corn and palm oils. FTIR spectroscopy combined with PLSR has been optimized and developed for authentication analysis by quantifying corn oil (CO) and soybean oil (SO) as oil adulterants in NSO. Based on the optimization procedure, quantitative analysis of NSO adulterated with CO was carried out using 2nd FTIR spectra at combined wavenumbers of 2977–3028, 1666–1739, and 740–1446 cm^−1^ providing R^2^ of 0.9984 and RMSEC of 1.34% *v*/*v*. NSO adulterated with SO is successfully determined at the combined wavenumbers of 2985–3024 and 752–1755 cm^−1^ using 1st derivative FTIR spectra, with R^2^ and RMSEC obtained being of 0.9970 and 0.47% *v*/*v*, respectively. In addition, 2nd FTIR spectra at the combined wavenumbers of 2977–3028, 1666–1739, and 740–1446 cm^−1^ were used for quantification of NSO adulterated with CO and SO in ternary mixture, having an R^2^ of 0.9993 and RMSEC value of 0.86% *v*/*v* [85].

### 4.7. Cod Liver Oil

Cod liver oil (CLO) is one of the valuable fish oils with a high price value due to the high contents of vitamin A and vitamin D as well as omega fatty acids of EPA and DHA. Common vegetable oils have the potential to be used as CLO’s adulterant due to the similar color between CLO and vegetable oils, so that visual detection is very difficult. Therefore, FTIR spectroscopy is mostly reported for authentication analysis. Rohman et al. [68] have reported the application of FTIR spectroscopy combined with chemometrics of multivariate calibration (PLSR and PCR) for quantitative analysis of canola oil (CaO), corn oil (CO), soybean oil (SO), and walnut oil (WO) in CLO, while LDA was used for classification or discrimination between CLO and CLO mixed with CaO, CO, SO, and WO. Some wavenumber regions were optimized, relying on the highest R^2^ values for the correlation between the actual values of adulterants and the predicted values as well as the lowest values of errors (RMSEC, RMSECV, and RMSEP). The combined 1/λ regions of 1112–1083, 1277–1197, and 1460–1450 cm^−1^ were used for the quantification of CaO; 1480–1375 and 2870–2820 cm^−1^ for CO; the combined region of 1113–1099, 1273–1211, and 3031–3002 cm^−1^ for SO; the combined region of 1117–1083 and 1257–1211 cm^−1^ for WO. PLS with FTIR normal spectra was successfully used for quantitative analysis of oil adulterants with R^2^ > 0.99 and RMSEC in the range of 0.04–0.82% (*v*/*v*). RMSEP values were of 1.75% (CaO), 1.39% (CO), 1.35% (SO), and 1.37% (*v*/*v*) (WO). LDA using the same wavenumbers region is successfully applied for discrimination of CLO and CLO adulterated with these vegetable oils with accuracy levels of 100%, meaning that no misclassified groups were reported.

### 4.8. Grape Seed Oil

Grape seed oil (GSO) is one of the highest quality edible oils obtained from the seeds of grapes in the pomace left over from juice and wine production. GSO contains high levels of beneficial bioactive compounds toward human health, including tocopherols and tocotrienols, polyphenols, flavonoids, tannins, and essential fatty acids; as a consequence, GSO had an expensive price [86]. The adulteration of GSO with the lower price of oils, namely refined soybean oils (SO), has been reported, and FTIR spectroscopy combined with multivariate data analysis (chemometrics) was used for the detection of such adulteration. Thirty-three pure oils and 99 blends of GSO-SO were analyzed using the ATR sampling technique, and the spectra were subjected to chemometrics of PCA and SIMCA for classification as well as PLSR for quantitative analysis. After optimization in terms of selecting wavenumbers capable of providing the desired purposes, PCA using combined wavenumber regions of band 1147–1127 (due to C-O stretching), 1127–1106 (C-O stretching), and 802–650 cm^−1^ (due to C-C, O-H bending) was applied for differentiation between GSO and other edible oils. In addition, SIMCA using the same wavenumbers region used in PCA provided an excellent classification for pure GSO and GSO adulterated with SO with classification limits reported was below 5%. Quantitative analysis of SO in GSO was assisted by PLSR, resulting in an R^2^ of >0.99 with minimum RMSEC and RMSECV. The RMSEC values were in the range 0.59–2.09%, while RMSECV values were in the range 0.92–5.60%, with detection limits of SO of 0.59% [69].

### 4.9. Pomegranate Seed Oils

Pomegranate oil (PGO) is a by-product from the extraction of seed of pomegranate fruit. PRO has been reported as a good source of certain pharmaceutical and nutraceutical compounds, including polyunsaturated fatty acids especially punicic acid (18:3 cis 9, trans 11, cis 13), an isomer of conjugated linolenic acid, tocopherols, phytosterols, and squalene [87]. Several health benefits of PRO were reported namely antidiabetic, antiproliferative, antiobesity, and anticarcinogenic effects [88]. PRO is considered as a valuable product economically, therefore PRO has been subjected to adulteration by mixing with cheaper and/or lower quality oils. PRO has been modelled to be adulterated with sunflower oil (SFO). The selection of SFO as oil adulterant was based on the facts; namely, its ease of availability around the world, its low price, and ease of mixing with PRO without introducing any noticeable flavor. FTIR spectra of pure PRO and PRO adulterated with SFO were scanned using ATR at mid IR region (4000–650 cm^−1^) with a resolution of 4 cm^−1^, number of scans of 64, and scanning speed of 1 cm/s. The chemometrics of OPLS-DA and PLS were used for classification of pure PRO and PRO adulterated with SFO and for the prediction of SFO levels in SFO, respectively. Using absorbance values at wavenumbers of 2924, 2852, 1723, selected fingerprint region (1464–983 cm^−1^), and 723 cm^−1^, OPLS-DA using the first and second latent variables (LVs) could classify pure PRO and PRO adulterated with SFO with clear separation. PLSR using second derivative spectra with three LVs could predict the levels of SFO with R2 value for the correlation between actual versus predicted values of 0.99 in calibration and prediction models with values of RMSEC of 0.57, RMSECV of 1.51, RMSEP of 1.42, and RPD of 12.48%, respectively. Using this calibration, the possible detection limit is 1%, which is a quite satisfactory threshold value in authentication analysis [89].

### 4.10. Pumpkin Seed Oil

Pumpkin seed oil (PSO) is popular as a functional food oil because of some bioactive components believed to be responsible for its biological activities such as phenolics, tocopherols, and other minor components. Some beneficial effects of PSO to human health have been reported such as anticancer, antioxidants, retardation of hypertension progression, and alleviation of diabetes mellitus [90]. Therefore, PSO is a target of adulteration with low-quality oils. Some vegetable oils and others have been subjected to PCA to search which oils have the similar FTIR spectra with PSO, and based on score plot PC1 and PC2, sesame oil (SeO) has a similar profile as PSO. FTIR spectroscopy combined with PLSR and DA have been applied for authentication of PSO from SeO. All FTIR spectra were subjected to derivatization, and based on higher R^2^ and lowest errors, 1st derivative spectra using absorbance values at wavenumbers of 1800–663 cm^−1^ as variables were used during PLSR and DA. The relationship between actual values of PSO in SeO with predicted values resulted good accuracy, as indicated by high R^2^ values of 0.9998 and 0.9994 in calibration and validation models. The model also revealed good precision, as indicated by low RMSEC and RMSEP values, each recorded as 0.003 and 0.006%, respectively. The chemometrics of DA using 10 PCs could clearly discriminate PSO and PSO adulterated with SeO with accuracy levels of 100%. Therefore, it can be concluded that FTIR spectroscopy in combination with chemometrics could be an effective tool for authentication analysis of PSO from SeO [91].

### 4.11. Palm Oil

Portable near infrared (NIR) spectroscopy has been used for authentication analysis of palm oil from lard (pork fat). The effect of path length of MicroNIR toward spectral measurement was evaluated by performing two scanning modes (transflectance and transmission). NIR spectra using both scanning modes were measured at wavelength 800–1700 nm. Spectral data were split using Kennard–Stone algorithm to be subjected with chemometrics classification of SIMCA and quantification using PLSR. The variable selection was performed using cumulative adaptive reweighted sampling (CARS). SIMCA could classify pure palm oil and palm oil adulterated with lard with accuracy level of higher 0.95, using absorbance values at wavelength 800–1700 nm for both modes. PLSR could predict the level of lard in palm oil with R^2^ values of 0.9987 and 0.9994 using transflectance and transmission spectra, respectively. RMSEC obtained were of 0.5931 (transflectance) and 0.6703 (transmission). The equation corelating between actual values of lard (*x*-axis) and predicted values using NIR-PLSR were:

*y* = 0.9987*x* + 0.02032 (transflectance) (3)

*y* = 0.9994*x* + 0.01024 (transmission) (4)

This technique can be applied as an on-site application for the detection of non-halal fats (lard) in palm oil for halal authentication [92].

### 4.12. Passion Fruit Oil (PFO)

PFO is an oil extracted from fruit seeds of *Passiflora edulis* mainly used during making juices and desserts. PFO contained polyunsaturated fatty acids and other bioactive compounds including sterols, vitamin E, and carotenoids, having potential health-promoting properties such as antioxidant, antitumor, and antibacterial activities. PFO has a high price in the market (approximately USD 50–200 per liter), thus PFO is subjected to adulteration by blending it with pracaxi oil, sunflower oil, and olive oil [93]. PCA and PLSR using the variable of absorbance values of FTIR spectra have been applied for authentication of origins of PFO and quantification of sunflower (SFO) in PFO. PCA using absorbance values at wavenumbers of 4000–650 cm^−1^ could classify PFO from five different origins and pure PFO. PCA also successfully classified PFO and PFO adulterated with SFO with different levels: 0.4, 5%, and other concentrations. PLSR using whole mid IR spectra facilitated the quantification of SFO added to PFO with R^2^ > 0.99 [94].

### 4.13. Argan Oil

Argan oil (ARO) is oil extracted from fruit kernels of the argan tree (*Argania spinosa*) and is traditionally used by the Berber population to treat hypercholesterolemia and atherosclerosis and to act as hepatoprotective and choleretic. ARO is also used as good oil component in cosmetics products capable of curing skin pimples, juvenile acne, and chicken pox pustules and reducing the rate of wrinkle appearances [95]. ARO can be target of adulteration, because ARO has a high price in the fats and oils industry. NIRS and visible spectroscopies were developed and validated for authentication analysis of ARO from cheaper oils marked with AO1 and AO2. Pure ARO was added with adulterants in the levels of 0–30%. Visible and NIR spectra were scanned at wavelength of 500–1000 and 1000–1700 nm, respectively. PCA and PLSR were applied for classification and quantification of adulterants in ARO. The classification of pure ARO and ARO adulterated with AO1 and AO2 was performed using absorbance values at 500–1000 and 1000–1700 nm, which resulted in clear separation between ARO adulterated with AO1 and ARO adulterated with AO2. For the quantitative analysis of adulterants, absorbance values at 500–1000 nm provided the best model. The correlation between actual values of ARO and predicted values revealed a good relationship with the coefficient of correlation (*R*-value) of 0.923 and 0.907 in calibration and prediction, respectively. RMSEC and RMSEP values obtained were 3.22% and 4.67%, respectively [96].

### 4.14. Wheat Germ Oil

Wheat germ oil (WGO) is one of the functional edible oils extracted from germ parts of wheat (*Triticum aestivum* L.). Some fat-soluble bioactive compounds have been contained in WGO such as tocopherols or vitamin E, phytosterols, carotenoids, policosanols, thiamin, riboflavin, and some polyunsaturated fatty acids (PUFA). These bioactive compounds are responsible for its biological activities including antioxidants, reducing cholesterol levels, improving physical endurance, and retarding the effects of aging [97]. FTIR spectroscopy combined with chemometrics of classification (PCA and LDA) and multivariate calibration of PLSR was reported for authentication of WGO from soybean oil (SBO) and sunflower oil (SFO). PCA using absorbance values at the whole FTIR spectral region (4000–650 cm^−1^) could classify WGO from other vegetable oils including SBO and SFO by exploiting two PCs (PC_1_ =  52%, PC_2_ =  28%). LDA classification methods using the same variables used in PCA provided good discrimination results of WGO samples and those adulterated with SBO and SFO. Quantification of adulterants (SFO and SBO) in WGO was performed using PLSR applying absorbance values at 4000–650 cm^−1^. A high correlation was obtained during modelling between actual values of SFO and SBO with predicted values with the slope values close to 1 and R^2^ values were of 0.9431 and 0.9260 for binary mixtures of WGO–SFO and WGO–SBO sets, respectively. The precision of method as evaluated by RMSEC and RMSECV was acceptable. The values of RMSEC were in the range 0.56–1.98% and RMSECV in the range 0.68–4.46. FTIR spectroscopy coupled with chemometrics provided rapid detections of SFO and SBO in cold-pressed WGO [98].

### 4.15. Mustard Oil

Mustard oil (MO) is one of edible oil sources in India and is considered as high valuable oil due to high levels of mono-unsaturated fatty acids (MUFA) and poly-unsaturated fatty acids (PUFA), including the omega-3 and omega-6 fatty acids needed to provide a balanced and healthy diet [74,99]. FTIR spectroscopy combined with chemometrics of PCA, LDA, PCR, and PLSR was used for classification and quantification of MO adulteration with argemone oil (AO). Spectral treatments including derivatization were used. PCA using combined region of 3050–2750 and 1800–500 cm^−1^ could make discrimination of MO from AO, while LDA using the same wavenumbers region could be applied for classification between MO and MO adulterated with AO. PCR and PLSR were compared, and finally, PLSR using the first derivative spectral at region of 1800–500 cm^−1^ showed the best calibration model with high precision as indicated by low value of relative prediction error of 0.033% and RMSEP of 0.2% vol/vol as well high correlation as indicated by high R^2^ of >0.999. The lowest detected percentage of AO in MO was 1% *v*/*v* [72].

Recently, ATR-FTIR spectroscopy combined with chemometrics of PCA and LDA for classification and multivariate calibrations (PCR and PLSR) for quantification has been applied for detection and quantitative analysis of fried mustard oil (FMO) as an adulterant in pure mustard oil (PMO) in the range of 0.5–50% *v*/*v* of FMO in PMO. PCA using both PC1 and PC2 corresponding to variances of 93% and 4% can discriminate between PMO and PMO adulterated with FMO in binary admixtures. In addition, LDA showed the discrimination between two groups with an accuracy level of 100% either in calibration/training or cross validation. For quantitative analysis, FTIR spectra were subjected to several spectral treatments including smoothening and Savitzky–Golay derivatization before being subjected to multivariate calibrations (PCR and PLSR). PLS-R using 2nd derivative spectra at optimized wavenumber regions of 1260–1080 cm^−1^ showed the best results for prediction of adulterant (FMO) levels providing the highest R^2^ (0.999) with a residual predictive deviation (RPD) value of 31.91, low RMSEC of 0.53% *v*/*v*, and relative prediction error of 3.37%. The developed method could detect the adulteration level as low as 0.5% *v*/*v* [35].

### 4.16. Butter

The adulteration of butter fat with margarine was analyzed using temperature-controlled attenuated total reflectance-mid-infrared (ATR-MIR) spectroscopy combined with chemometrics. Commercial samples of butter fat were adulterated with margarine fat at levels ranging from 0 to 100% (*v*/*v*). In order to resolve the overlapped peaks, the Savitzky–Golay second derivative transformation of MIR spectra was used during quantification of adulterants. Absorbance values at combined wavenumbers region of 3040–3000 and 1500–1000 cm^−1^ assisted by PLSR could be used for prediction of adulterant levels. The correlation coefficient (*R*-value) for the relationship between actual and predicted values were >0.99 with standard error of cross-validation (SECV) of <1.2% (*v*/*v*). SIMCA models using 2nd derivative spectra at combined region 3040–2800 and 1800 to 900 cm^−1^ could classify authentic butter, regular margarine fat, and light margarine fat [100].

FTIR spectroscopy combined with chemometric classification of PCA and PLS-DA was successfully used for the authentication of butter from different origins. The butter samples produced in Morocco in areas of Fkih Ben Saleh, Kssiba and Kalaa Sraghna were discriminated and classified. PCA using mean centering pre-processing at wavenumbers of 3000–600 cm^−1^ could classify butter according to its origin exploiting PC1 and PC2, which corresponded to variances of 74% (PC1) and 14% (PC2). PLSDA using the same wavenumbers was also capable of discriminating among groups (authentic and adulterated) with accuracy levels of 100% [101].

### 4.17. Ghee

The presence of adulterant of body pig fat (BPF) in pure ghee was evaluated by normal FTIR spectra combined with chemometrics. Pure ghee and that adulterated with BPF was classified using SIMCA using wavenumbers of combined 1/λ of 3030–2785, 1786–1680, and 1490–919 cm^−1^ resulting in the accuracy level of classification of >90%. In addition, some 1/λ regions were optimized for quantitative analysis of BPF in ghee, namely at 1/λ 3030–2785, 1786–1680, and 1490–919 cm^−1^. Finally, wavenumbers of 3030–2785 cm^−1^ were used for the quantification of BPF with R^2^ value of 0.998 (in calibration and validation models) with a RMSEC of 1.43% and RMSECV of 1.48%. The high level of R^2^ and low values of RMSEC and RMSECV indicated that FTIR spectra combined with suitable chemometrics are a reliable method for the authentication analysis of ghee with high correlation and precision [67].

The adulteration may take place by addition of used frying oil (UFO) into vegetable oils typically used as frying oils such as corn (CO), peanut (PEO), rapeseed (RSO), and soybean oil (SO). UFO was collected from the sales stand in the local street, mainly composed from SO. FTIR spectroscopy combined with chemometrics of cluster analysis and discriminant analysis was used for classification. In addition, linear regression was used for quantification. Cluster analysis was exploited for classification of samples in calibration sets. CO and PEO were classified into four categories, while RSO and SO were classified into five categories. DA was used for qualitative analysis of validation sets. The adulteration levels of UFO added into vegetable oils were 1–90%. Quantitative analysis of the adulterant (UFO) was facilitated by liner regression using ratio of peak area of band 19 (1/λ 968 cm^−1^) and band 20 (1/λ 914 cm^−1^) and a wavenumber shift of band 19 (1/λ 914 cm^−1^). The R^2^ values for correlation between actual values of UFO in vegetable oils and predicted values were higher than 0.99 for all samples. The limits of detection (LODs) of UFO in CO, PEO, RSO, and SO using the variable of area ratio were 6.6, 7.2, 5.5, 3.6%, respectively; while using the variable of wavenumber shift, LOD values reported were 8.1, 9.0, 6.9, and 5.6%, for CO, PEO, RSO, and SO, respectively [102].

## 5. Authentication of Fats

**Figure 1:**
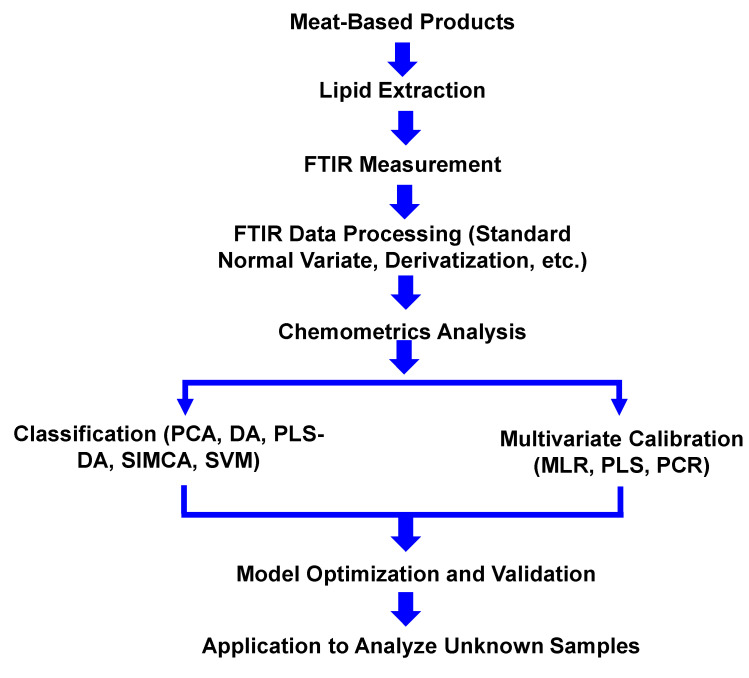
The analytical procedure for analysis of meat-based food products using FTIR spectroscopy method.

FTIR spectroscopy combined with chemometrics is an ideal technique for analysis of fats including fats extracted from meat in food products. Meat-based food products containing non-halal meats were extracted using appropriate extraction techniques to extract lipids, and lipids obtained were subjected to instrumental analyses [16]. Food products containing meat were hydrolyzed and extracted their fat content for FTIR analysis. Schematically, the analytical procedure for analysis of meat-based food products using FTIR spectroscopy combined with chemometrics is depicted in Figure 1.

Table 2 presents the use of FTIR spectroscopy in combination with chemometrics for the analysis of fats in food products.

### 5.1. Meatball

Meatball is one of the most preferred meat-based products particularly in Indonesia and Malaysia. Commonly, meatball is formulated using beef meatball; however, because of its expensive price, it is often adulterated with other lower price meats, for instance pork, dog meat, wild boar meat, as well as rat meat. The presence of these meats is prohibited especially for Muslims, because it is categorized as non-halal substances. The presence of rat meat in beef meatball has been detected and quantified using FTIR spectroscopy and chemometrics of PCA and PLS. Meatball samples were sliced into small pieces and homogenized using a blender. The fats were extracted using hexane employing Soxhlet method. The extracts were evaporated, and the obtained fats were used for FTIR measurement using ATR technique. PCA using wavenumber of 1000–750 cm^−1^ was perfectly applied for differentiating between beef meatball and rat meatball. Chemometrics of PLS successfully predicted the concentration of rat meat in beef meatball measured using the fats content. The same wavenumber region was used to create the PLS model resulting in R^2^ of 0.993 for calibration and 0.994 for the validation model, whereas low RMSEC value (1.79%) and RMSEP value (0.90%) were obtained, indicating the good correlation and precision of the PLS model [106].

FTIR spectroscopy in combination with chemometrics of PCA and PLS has also been applied for the analysis of wild boar meat in beef meatball. Fats were extracted using the Soxhlet method employing n-hexane as the solvent. PCA using wavenumber of 1250–1000 cm^−1^ perfectly classified between meatball from wild boar meat and meatball from beef. The presence of wild boar meat in beef meatball was successfully quantified using chemometrics of PLS employing the same wavenumber used for PCA. The PLS model showed high R^2^ either in the calibration (0.998) or in the validation (0.986) model with a low value of RMSEC (2%) and RMSEP (5.84%) [115].

### 5.2. Sausage

Sausage is another meat-based product that is usually made from beef or chicken meat. Sausage also has the potential to be adulterated with other meats having a lower price for economic reasons. The presence of other meats is obviously difficult to be detected using visual inspection, because it has been mixed with other ingredients. The presence of lard in beef sausage has been investigated using FTIR spectroscopy and chemometrics. Lard was extracted from sausage using the Soxhlet method employing n-hexane as the solvent. After evaporating the solvent, the obtained fats were used for FTIR measurement. Chemometrics of PCA using wavenumber 1200–1000 cm^−1^ successfully differentiated between beef sausage and beef sausage containing lard. The concentration of lard in beef sausage was also successfully predicted using chemometrics of PLS at the same wavenumber. The PLS model resulted in R^2^ of 0.985 with RMSEC of 2.094%, RMSEP of 477%, and RMSECV of 5.12% [116].

FTIR spectroscopy and chemometrics has also been used for analysis of dog meat in beef sausage. Dog meat is widely available and often used by unethical producers to be mixed in meat-based food products. Dog fat was extracted from beef sausage prior to FTIR measurement. The presence of dog fat was successfully classified using chemometrics of PCA created using a wavenumber of 1124–688 cm^−1^. Chemometrics of PLS were successfully applied for the quantification of dog fat in beef sausage providing high R^2^ values (more than 0.999) and low RMSEC (0.30%) and RMSEP (0.05%) [117]. The presence of rat meat in beef sausage has also been investigated using FTIR spectroscopy and chemometrics. Fats were extracted using three different lipid extraction methods, namely Bligh and Dyer, Folch, and Soxhlet extraction methods from beef sausage, and the FTIR spectra were recorded using FTIR-ATR spectrophotometer using a wavenumber of 4000–650 cm^−1^. Chemometrics of PCA could completely separate between beef sausage and rat meat sausage extracted using three different methods at wavenumber of 1800–750 cm^−1^. Whereas, chemometrics of PLS were also successfully employed for predicting rat meat concentration in beef sausage with a high value of R^2^ and low value of RMSEP and RMSEC, indicating that FTIR and chemometrics could be used for the analysis of rat meat in beef sausage [108].

### 5.3. “Rambak” Cracker

Rambak is one of Indonesian traditional food products usually made from cow and buffalo skin. Rambak has wide applications to be served in combination with various food products, and it is widely spread especially in the Indonesian and Malaysian markets [85,118]. Several sources are available for making rambak including pig skin instead of cow and buffalo skin. It is very challenging to differentiate rambak made from pig skin and others, because the final products of rambak made from different sources of animal skin are very similar. The presence of pig skin in buffalo skin rambak has been successfully detected using FTIR spectroscopy and chemometrics of PCA and PLS. The Soxhlet extraction method was used for lipid extraction from rambak samples using hexane as the solvent. The acquisition of lipid spectra was performed using FTIR-ATR spectrophotometer at the wavenumber of 4000–650 cm^−1^. Chemometrics of PCA performed at wavenumber of 1200–1000 cm^−1^ demonstrated a great result for differentiation between rambak containing pig skin and buffalo skin. PCA could identify the presence of pig skin and buffalo skin in rambak crackers measured by their lipid content. Meanwhile, chemometrics regression of PLS using eight of number factors could predict lard concentration in buffalo skin rambak with a good correlation between actual concentration and FTIR predicted concentration. The PLS model built using the same wavenumber used for PCA resulted in a high value of R^2^ for the calibration (0.961) and validation (0.994) model with a low value of RMSEC (2.56%) and RMSEP (1.10) [112].

Another study on investigating the adulteration of rambak cracker has also been carried out using FTIR spectroscopy. Chemometrics of PCA and PLS were applied for the detection of pig skin in rambak made from cow skin. Both of PCA and PLS models were carried out at the wavenumber of 1200–1000 cm^−1^. Rambak made from pure cow skin and contaminated with pig skin could be completely separated using PCA. Moreover, several commercial samples were also detected, and the result suggested that the commercial samples did not contain pig skin. Quantification of lard in rambak has been successfully performed using chemometrics of PLS, resulting in a good correlation between actual and FTIR-predicted concentration of lard in rambak. The coefficient of determination (R^2^) showed a good performance of the PLS model accounting for 0.946 for the calibration and 0.997 for the validation model. The low values of RMSEC (2.77%) and RMSEP (2.77%) demonstrated the high precision of the PLS model. Therefore, FTIR spectroscopy combined with chemometrics could be a rapid and reliable method for detection of adulteration in rambak cracker products [113].

### 5.4. Beef Jerky (Dendeng)

Beef jerky also known as dendeng, is usually made from beef, and it is one of the preferred foods in Indonesia and Malaysia. The ingredients of beef jerky are beef, brown sugar, particular spices, and salt. The obtained jerky was then processed in an oven for approximately 6 h. The beef used in making jerky is susceptible to being adulterated with pork, because pork is obviously cheaper than beef [119]. Moreover, it is very challenging to detect the presence of pork in cooked beef jerky just by visual inspection, because the appearance is very similar, so that it is very difficult to differentiate. FTIR spectroscopy and chemometrics of classification have been used for authentication of beef jerky adulterated with pork. For FTIR measurement, the jerky samples were prepared by slicing into small pieces and homogenized using a blender. The obtained powder was measured using FTIR spectrophotometer at 4000–700 cm^−1^. Chemometrics of LDA, soft independent modeling class analogy (SIMCA), and support vector machine (SVM) at different wavenumber regions were used for classification. The best model was obtained using LDA employing the fingerprint wavenumber region (1500–600 cm^−1^). The LDA model could predict all samples accurately (100% of accuracy). Therefore, it is suggested that FTIR and chemometrics could be good combination method for analysis of pork in beef jerky [114].

### 5.5. Milk Fat

Milk fat is considered as one the high nutrition fat products due to its functional properties. It contains a lot of nutrition such as lipid soluble vitamin, especially vitamin D, and several fatty acids, which are beneficial for health. Adulteration with lower quality fats is often performed to gain more benefits. FTIR spectroscopy combined with chemometrics of discriminant analysis (DA) using normal spectra at the wavenumber of 3098–669 cm^−1^ has been successfully used for discrimination between pure bovine milk fat with adulterated bovine milk fat with lard. All of the adulterated samples were clearly separated from pure bovine milk fat even in the lowest adulterant concentration (5% *w*/*w*). Meanwhile, chemometrics of PLS demonstrated a good model to predict the concentration of lard in the mixtures with bovine milk fat. The PLS model was created using the first derivative spectra at wavenumber combination of 3033–2770 and 1510–692 cm^−1^. The obtained R^2^ was higher than 0.99 both in the calibration and validation model, whereas the value of RMSEC and RMSEP was 0.631 and 1.94, respectively. It suggested that FTIR and chemometrics are suitable methods for authentication analysis of milk fat [120].

## 6. Conclusions

Research and innovation on the development of analytical method capable of detecting the adulteration practice in fats and oils have grown rapidly. Due to its nature as fingerprint analytical technique, FTIR spectroscopy and other vibrational spectroscopic techniques have been commonly used for food compositional analysis and authentication. Combined with suitable chemometrics techniques, FTIR spectroscopy using attenuated total reflectance and employing suitable variables (absorbance values at certain wavenumbers) are ideal techniques for authentication of fats and oils due to their simplicity and their being user friendly without extensive analytical processes. The combination of FTIR and chemometrics has emerged as powerful in authentication analysis for the analysis of fats and oils, as indicated in this review. In the future, this method must be standardized to be applied in the quality control laboratories of fats and oils.

## Figures and Tables

**Table 1:** The application of FTIR spectroscopy combined with chemometrics for authentication analysis of oils.

Adulterated Fats and Oils	Adulterants	Wavenumbers (1/λ) Region	Spectral Treatment	Chemometrics Techniques	Remarks	References
Extra virgin olive oil (EVOO)	Corn oil (CO) and sunflower oil (SFO)	3027–3000, 1076–860, and 790–698 cm^−1^ for CO and 3012–3000 cm^−1^ for SFO	Derivatization	DA for classification and PLS for quantification	Classification of authentic EVOO (extra virgin olive oil) and adulterated EVOO with CO and SFO was successfully performed using DA with no misclassification reported. PLS using normal spectra resulted high value of R^2^ (>0.99) with RMSEC of 0.404% and RMSEP of 1.13%, whereas the presence of SFO could be quantified using PLS employing first derivative spectra with R^2^ more than 0.99 and low value of RMSEC (0.035%) and RMSEP (2.02%).	[50]
	Soybean oil (SB) and sunflower (SF) oil	3035–670 cm^−1^	Mean centering	PLS for quantification and PLS-DA for classification	PLS could predict the concentration of SB and SF in EVOO with high R^2^ of calibration (0.991), RMSEC of 0.57 and high R^2^ of prediction (0.997), and RMSEP of 0.41. Chemometrics of PLS-DA could classify EVOO and adulterated EVOO with SB and SF accurately.	[51]
	Canola oil (CaO)	3028–2985 and 1200–987 cm^−1^	No spectral treatment	PLS for quantification and DA for classification	PLS could be used for quantitative analysis of CaO in EVOO with high R^2^ value (>0.99) and low RMSEC value (0.108). DA completely separated between authentic EVOO and adulterated EVOO with CaO with no misclassification.	[47]
	Grapeseed oil (GSO) and walnut oil (WO)	3018–3002 and 1200–1000 cm^−1^ for GSO and 3029–2954 and 1125–667 cm^−1^ for WO	No spectral treatment	DA for classification and PLS for quantification	DA perfectly classified between authentic EVOO and adulterated EVOO with GSO and WO with no misclassification reported. PLS using normal spectra resulted high value of R^2^ both calibration (0.999) and validation (0.994) model with low value of RMSEC (0.38%) and RMSEP (1.32%) for quantification of GSO, whereas PLS using normal spectra could be used for quantification of WO with R^2^ of calibration and validation more than 0.99 and low value of RMSEC (0.101%) and RMSEP (0.934%).	[52]
Virgin coconut oil (VCO)	Corn oil (CO) and sunflower oil (SFO)	3028–2983, 2947–1887, and 1685–868 cm^−1^, (VCO mixed with CO) and combined wavenumbers of 3030–2980 and 1300–1000 cm^−1^ (VCO mixed with SFO)	Derivatization	PLSR for quantification and DA for classification	DA could classify VCO and VCO adulterated with CO and SFO without any misclassification reported (accuracy level 100%). PLSR at wavenumbers of 858–705, 943–863, 1392–983, and 3027–2983 cm^−1^ was used for quantification of CO in VCO resulting in R^2^ of 0.999, RMSEC of 0.866%, and RMSEP of 0.990%. SFO in VCO was quantified using wavenumbers of 1685–686, 2946–1887, and 3027–2983 cm^−1^ resulting in R^2^ of 0.999, RMSEC of 0.374%, and RMSEP of 1.06%.	[53]
	Grape seed oil (GSO) and soybean oil (SO)	Combined wavenumbers of 1200–900 and 3027–2985 (GSO in VCO) and 200–1000 and 3025–2995 cm^−1^ (SO in VCO)	Mean centering and derivatization	PLSR and PCR for quantification and DA for classification	DA was successfully used for classification of VCO and VCO added with adulterants of GSO and SO. PLSR at these wavenumbers could quantify the levels of adulterants (SO and GSO) with R^2^ of 0.994–0.998, RMSEC of 0.007–0.268%, and RMSEP of 1.32–1.70%.	[54]
	Canola oil (CaO)	Wavenumbers of 1200–900 and 3027–2985 cm^−1^	Derivatization	PLSR and PCR for quantification and DA for classification	DA was able to discriminate VCO and that adulterated with CaO. PLSR using normal spectra was preferred more than PCR for quantification of CaO in VCO with R^2^ of 0.998 and 0.996 in calibration and validation models, RMSEC of 0.392%, and RMSEP of 2.57%.	[55]
	Quantification of VCO in binary mixture with palm oil (PO)	Combined wavenumbers of 1120–1105 and 965–960 cm^−1^	Normal spectra	PLSR and PCR	PLSR was able to quantify VCO with R^2^ and RMSEC values were of 0.9996 and 0.494, respectively.	[56]
	Analysis of palm oil as VCO’s adulterant	Combined wavenumbers of 3010–3000, 1660–1650, and 1120–1105 cm^−1^	Derivatization	PLSR and DA	PLSR showed good relationship between actual and FTIR-predicted values of PO with R^2^ of 0.999 and standard error of calibration of 0.533. The value of R^2^ during cross validation was 0.996, and standard error of prediction was 0.953. DA using 7 PCs was able to classify pure VCO and that adulterated with PO.	[57]
	Palm kernel oil (PKO)	Whole IR region (4000–650 cm^−1^)	No spectral treatment	PLSR and DA	PLSR could quantify PKO using 10 PCs with detection limit of 1%. DA could classify VCO and VCO mixed with other vegetable oils (walnut, extra virgin olive, soybean, sunflower, grapeseed, sesame, canola, and corn oils).	[58]
	Lard (LD)	Combined wavenumbers of 3020–3000 cm^−1^ and 1120–1000 cm^−1^	No spectral treatment	PLSR and DA	PLSR could predict LD contents in VCO with R^2^ of 0.9990. DA can classify VCO and that adulterated with LD with an accuracy level of 100%.	[59]
Red fruit oil (RFO)	Sunflower oil (SFO) and palm oil (PO)	1200–1000 cm^−^^1^ (SFO in RFO), 1780–1680 cm^−^^1^ (PO in RFO)	Savitzky–Golay derivatives	PCA, PLSR	PCA is successfully used to identify PO and SFO as adulterants in RFO. PLSR using normal FTIR spectra at optimized wavenumbers could quantify oil adulterants (PO and SFO) in RFO with R^2^ > 0.99, RMSEC of 1.0011 (PO), and R^2^ of 0.9956 and RMSEC of 1.4187% (SFO).	[60]
	Corn oil (CO) and soybean oil (SO)	Combined frequency region of 1800–1600 and 1200–800 cm^−1^	No spectral treatment (using normal spectra)	PLSR	The R^2^ value of 0.999 and RMSEC of 0.987% (*v*/*v*) were obtained during modelling the relationship between actual values and predicted values of CO and R^2^ and RMSEC values of 0.997 and 1.195% obtained for the quantification of SO.	[61]
	Corn oil (CO) and soybean oil (SO) in ternary mixture with RFO	4000–650 cm^−1^	Derivatization	PLSR	The simultaneous analysis was successfully performed with R^2^ values obtained for the relationship between actual and FTIR predicted values of RFO, CO, and SO were 0.9863, 0.9276, and 0.9693, respectively. RMSEC values obtained were 1.59, 1.72, and 1.60% (*v*/*v*) for RFO, CO, and SO, respectively.	[62]
Avocado oil (AVO)	Soybean oil (SO) and corn oil (CO)	1427–779 cm^−1^ (SO in AVO) and combined wavenumbers of 1477–721, 1728–1685, and 3035–2881 cm^−1^ (CO in AO)	Smoothing and derivation treatment	PLSR	FTIR normal spectra using PLSR were suitable for the quantification of SO in AO having R^2^ of 0.9994, RMSEC of 0.86%, and RMSEP of 0.88%. Meanwhile, R^2^ of 0.9994, RMSEC of 0.87%, and RMSEP of 0.52% were obtained for quantitative analysis of CO in AVO.	[63]
	Grape seed oil (GSO) and sesame oil (SeO)	Combined wavenumbers of 1006–902,1191–1091, and 1755–1654 cm^−1^ (GO in AVO) and 4000–650 cm^−1^ (SeO in binary mixture with AVO)	1st and 2nd derivatives	PLSR	FTIR spectra using PLSR could predict the levels of adulterants providing R^2^ of 0.9994 with low RMSEC of 0.86% (GSO in AVO); meanwhile, R^2^ of 0.9997 with RMSEC 0.73% *v*/*v* were obtained for analysis of SeO as adulterant in AVO. The validation models gave RMSEP values of 0.52% *v*/*v* (GSO) and 0.53% *v*/*v* (SeO).	[64]
Black seed cumin oil or *Nigella sativa* oil (NSO)	Grape seed oil (GSO)	Combined wavenumbers of 1114–1074, 1734–1382, and 3005–3030 cm^−1^		PLSR	PLSR using these wavenumbers could quantify GSO in NSO with R^2^ for the relationship between actual and FTIR predicted values of 0.981. RMSEC and RMSECV values were of 2.34% (*v*/*v*) and 2.37% (*v*/*v*), respectively.	[65]
	Walnut oil (WO) and sunflower oil (SFO)	4000–650 cm^−1^ (quantification), 3009–721 cm^−1^ (classification)	Derivatization	PLSR, PCA	PLSR at the whole region (4000–650 cm^−1^) is well suited for quantitative analysis of NSO in the binary mixture with WO and SFO. PCA using wavenumbers of 3009–721 cm^−1^ is successfully used for classification of NSO and NSO adulterated with SFO and WO.	[66]
Pure ghee	Pig body fat (PBF)	Combined 1/λ of 3030–2785, 1786–1680, and 1490–919 cm^−1^ (SIMCA) and at 3030–2785 cm^−1^ (PLS)	No spectral treatment (using normal spectra)	Quantification using PLS and classification with SIMCA and PCA	PLS could quantify PBF in pure ghee with R^2^ of 0.998 and RMSEC of 1.48%. SIMCA could classify pure and adulterated ghee with accuracy levels of >90%.	[67]
Cod liver oil	canola (CaO), corn (CO), soybean (SO), and walnut oils (WO)	Combined 1/λ 1112–1083, 1277–1197, and 1460–1450 cm^−1^ (CaO), 1480–1375 and 2870–2820 (CO), 1113–1099, 1273–1211, and 3031–3002 (SO), 1117–1083 and 1257–1211 cm^−1^ (WO)	Normal FTIR spectra, no spectral treatment	PLS for quantification, LDA for discrimination using the same wavenumbers regions	PLS with FTIR normal spectra is successfully used for quantitative analysis of oil adulterants with R^2^ > 0.99 and RMSEC in the range of 0.04–0.82% (*v*/*v*). RMSEP values were of 1.75% (CaO), 1.39% (CO), 1.35% (SO), and 1.37% (*v*/*v*) (WO). LDA could discriminate CLO and CLO adulterated with CaO, CO, SO, and WO.	[68]
Grape seed oil (GSO)	Soybean oil (SO)	The combined region of 1147–1127, 1127–1106, and 802–650 cm^−1^	Normal FTIR spectra, no spectral treatment	PCA and SIMCA (for classification), PLSR (for quantification)	SIMCA provided an excellent classification for pure GSO and GSO adulterated with SO with classification limits of <5%. Quantification of SO in GSO with PLSR resulted inR^2^ of >0.99. RMSEC values 0.59–2.09%, RMSECV of 0.92–5.60.	[69]
Pumpkin seed oil (PSO)	Sesame oil (SeO) and Rice Bran oil (RBO)	3100–2750 and 1500–663 cm^−1^	Derivative spectra	PLSR	PSO in ternary mixtures with RBO and SEO could be predicted with R^2^ > 0.99 along with RMSEC value of 0.0054% and RMSEP of 0.0179%	[70]
Pumpkin seed oil	Palm oil	Combined regions 3100–2750 and1500–663 cm^−1^	1st derivative spectra	PLSR and DA	R^2^ values obtained for correlation between actual versus predicted levels of PO were 0.9967 and 0.9906 in calibration and validation models. RMSEC and RMSEP were 0.0080 and 0.0152%. DA could classify two groups.	[71]
Mustard oil (MO)	Argemone oil (AO)	3050–2750 and 1800–500 cm^−1^	Derivative spectra	PCA and LDA (for classification), PCR and PLSR (for quantification)	PCA could make discrimination of MO from AO. DA could classify between MO and MO adulterated with AO. PLSR using the first derivative at 1800–500 cm^−1^ provided low value of RPE of 0.033% and RMSEP of 0.2% vol/vol, R^2^ of >0.999. The lowest detected percentage of AO in MO was 1% *v*/*v*.	[72]
Butter	Solid fraction of palm oil	3873–690 cm^−1^	Normal spectra	PLS	Detection limit 3% palm oil in butter and limit of quantification of 9.8%.	[73]
	Chicken fat (CF)	1200–1000 cm^−1^	Normal spectra	PLS	The levels of CF could be predicted with PLS with R^2^ of 0.98. RMSEC and RMSECV using 6 PCs were 2.08 and 4.33% *v*/*v*, respectively.	[74]
	Beef fat (BF)	1500–1000 cm^−1^	Normal spectra	PLS	BF in butter could be quantified with PLS with R^2^, RMSEC of 0.999 and 0.89% (*v*/*v*). Using 6 PCs, RMSEP obtained is 2.42% (*v*/*v*).	[75]
	Margarine (MR)	1400–800 cm^−1^	Normal spectra	PCA, SIMCA, PLSDA, PLSR	PCA made clustering of butter and MR. SIMCA could classify samples according to its group (authentic butter, MR, and butter adulterated with MR at 1–30%. PLS-DA could classify among groups with accuracy of 100%. PLS-R model (R^2^ = 0.84, RMSEP = 16.54%) was developed for quantification of MR in butter.	[76]
	Vegetable butter (3.8–40%) and of mashed potatoes (13–36%)	4000–2400 and 2300–600 cm^−1^	Second derivative	CA, PCA, LDA, SVM	PCA- LDA and SVM models using 2nd derivative spectra gave good classification according to its classes with accuracy of 97.22 and 100%, respectively.	[77]

RPE = relative prediction error; SVM = support vector machines (SVMs).

**Table 2:** The application of FTIR spectroscopy combined with chemometrics for authentication analysis of fats in meat-based food products.

Adulterated Meat/Food Products	Meat Adulterants	Wavenumbers (1/λ) Region	Extraction and Sampling Handling Technique	Spectral Treatment and Chemometrics	Remarks	References
Beef/meatball	Pork	1200–1000 cm^−1^	Soxhlet using hexane as an extraction solvent and fats extracted subjected to ATR	PLSR	PLSR using selected fingerprint regions of 1200–1000 cm^−1^ could predict pork fat (lard) extracted from meatball with R^2^ for the relationship between actual lard and FTIR-predicted lard was 0.999 with RMSEC of 0.442.	[103]
Beef/meatball	Pork	1200–1000 cm^−1^	Soxhlet using hexane as an extraction solvent and fats extracted subjected to ATR	PCA and PLSR	FTIR normal spectra were a fast technique for classification and quantification of lard extracted from pork in meatball. PCA is successful for the classification of samples containing pork and beef meatballs. PLSR could predict lard (lipid fraction obtained from meatballs containing pork) with R^2^ of 0.997 and standard error of calibration of 0.04%.	[104]
Beef/meatball	Pork through analysis of meatball broth	1018–1284 cm^−1^ (PLSR) and 1200–1000 cm^−1^ (PCA)	Meatball broth was taken and added with hexane to be subjected with LLC and fats obtained scanned using HATR	PLSR, PCA	Lard (pork fat) extracted from could be quantified with R^2^ and RMSEC values of 0.9975 and 1.34% (*v*/*v*). PCA also classifies meatballs containing beef and pork according to its group.	[105]
Beef/Meatball	Rat meat	1000–750 cm^−1^	Soxhlet using hexane as an extraction solvent. The fats obtained were subjected to HATR	PLSR, PCA	Rat meat could be quantified using PLSR resulting R^2^ for the relationship between actual values and FTIR-predicted values of 0.993 with RMSEC of 1.79%. PCA was successfully used for the classification of rat meat meatball and beef meatball.	[106]
Beef and chicken sausages	Pork	4000–400 cm^−1^	Ham sausage samples were grinded followed by preparation of KBr pellets.	Spectra were subjected to smoothing derivatives SNV. Classification using PLSDA	SNV can improve the classification accuracy of PLSDA. PLS-DA could classify halal (containing no pork) and non-halal (containing pork) sausages with sensitivity and specificity of 0.913 and 0.929 for PLSDA with SNV spectra, respectively.	[107]
Beef sausages	Rat meat	1800–750 cm^−1^	Sausages were extracted using three extraction methods namely, Bligh and Dyer, Folch, and Soxhlet. The lipids obtained were subjected to HATR	Spectra were subjected to mean centering and derivatization followed by PLSR	PCA using FTIR normal spectra could classify rat meat and beef lipids extracted by three extraction methods. For quantification of lipids extracted from beef meat sausages, R^2^ and RMSEC during PLS using Bligh and Dyer, Folch, and Soxhlet method were 0.945 and 2.73%; 0.991 and 1.73%; 0.992 and 1.69%, respectively. The values of R^2^ and RMSEP in validation were 0.458 and 18.90% (Folch) and 0.983 and 4.21% (Soxhlet).	[108]
Beef meatballs	Dog meat (DM)	Combined wavenumbers of 1782–1623 and 1485–659 cm^−1^	Lipids were extracted using Folch method and subjected to HATR measurement	FTIR spectra was subjected to detrending treatment followed by PLSR	DM in beef meatballs could be quantified by lipids extracted using PLSR. The values of R^2^ for correlation between the actual value of DM and FTIR predicted value was 0.993 in calibration model and 0.995 in validation model. RMSEC and RMSECV were 1.63 and 2.68%.	[109]
Beef meatballs	Dog meat (DM)	Combined wavenumbers of 1700–700 cm^−1^	The lipid fractions were extracted using Bligh–Dyer and Folch methods and then subjected to HATR.	No spectral treatment. Quantification was performed using PLSR and classification with PCA	PCA was capable of identifying and classifying DM in beef meatball. The values of R^2^, RMSEC, and RMSEP of lipids extracted using Folch higher than those of Bligh–Dyer.	[110]
Beef meat	Wild boar meat (WBM)	1250–1000 cm^−1^ (PLSR and PCA)	Lipids were extracted using Soxhlet method employing hexane as extracting solvent, and the obtained lipids were subjected to HATR	No spectral treatment. Quantification was performed using PLSR and classification with PCA	PLSR for the relationship between actual value of WBM and FTIR predicted value had equation of: predicted value = 0.9749 x actual value +1.4658 with R^2^ of 0.988 and RMSEC of 2.0%. PCA was successfully applied for the classification of wild boar meatball and beef meatball.	[111]
Buffalo skin	Rambak cracker containing Pig skin (PS)	1200–1000 cm^−1^ (PLSR and PCA)	Rambak crackers were extracted using Soxhlet method using hexane as extracting solvent, and the obtained lipids were subjected to HATR	No spectral treatment. Quantification was performed using PLSR and classification with PCA	The relationship between actual and predicted values of PS in rambak has R^2^ of 0.96, RMSEC of 2.56, and RMSEP of 1.10. The PCA models successfully classify types of buffalo skin, pig skin, and commercial rambak crackers.	[112]
Cow skin	Lard extracted from Rambak cracker containing pig skin (PS)	1200–1000 cm^−1^	Rambak crackers were extracted using Soxhlet method with hexane followed by FTIR spectra measurement	No spectral treatment. Quantification was performed using PLSR and classification with PCA	The relationship between actual value of lard extracted from PS in crackers and FTIR predicted value has R^2^ value of 0.946 with low errors in calibration and validation models. PCA can be successfully used for classification of rambak crackers with and without PS	[113]
Beef jerky	Pork	1500–600 cm^−1^	Jerky samples were sliced and ground into powder followed by FTIR spectra measurement	Classification using LDA, SIMCA, and SVM	Chemometrics of LDA demonstrated the best model for classification of beef jerky and pork jerky precisely and accurately without misclassification	[114]
